# *Limosilactobacillus fermentum* MG4295 Improves Hyperglycemia in High-Fat Diet-Induced Mice

**DOI:** 10.3390/foods11020231

**Published:** 2022-01-15

**Authors:** Ji Eun Kim, Ji Yeon Lee, Chang-Ho Kang

**Affiliations:** MEDIOGEN, Co., Ltd., Biovalley 1-ro, Jecheon-si 27159, Korea; jiun4141@gmail.com (J.E.K.); ljy341@naver.com (J.Y.L.)

**Keywords:** antidiabetic, antihyperglycemia, anti-NAFLD, metabolic disorder, probiotics

## Abstract

Hyperglycemia due to uncontrolled glucose regulation is widely known as cause of diabetes, non-alcoholic fatty liver disease (NAFLD), and other complications. NAFLD refers to a condition in which fat is excessively accumulated, whether inflamed or not, and has caused serious medical problems in recent years. The aim of this study was to explore the antihyperglycemia effects of *Limosilactobacillus fermentum* MG4295 (*L. fermentum* MG4295) in high-fat diet (HFD)-induced in vivo. We demonstrated the suitability of *L. fermentum* MG4295 as a probiotic by observing its stability, survivability, and proliferation under simulated gastrointestinal conditions, and safety, antibiotic susceptibility, hemolysis, and enzyme activity. The potential antihyperglycemic activity of *L. fermentum* MG4295 was investigated in an HFD and sugar-water-induced mouse model. Administration of this strain for 12 weeks showed an improved trend in glucose tolerance, insulin, alanine amino transferase, total cholesterol, low-density lipoprotein cholesterol, and glucagon-like peptide-1. Histopathological analysis revealed that *L. fermentum* MG4295 significantly reduced the histopathological scores of hepatic steatosis, inflammation, and hepatocellular hypertrophy in liver tissues and lipid content in adipose tissues. Administration of *L. fermentum* MG4295 upregulated *IRS-1, AKT,* and *GLUT4* and downregulated *G6Pc* and *PEPCK* expression in liver and/or muscle tissues. Our results suggest that *L. fermentum* MG4295 can improve hyperglycemia. Furthermore, it can be used as a dietary functional supplement to manage blood glucose.

## 1. Introduction

Hyperglycemia includes n several mechanisms of insulin resistance, enhanced glucose production, and high glucose loads [1]. These mechanisms have been reported to be closely related to diabetes mellitus (DM) and NAFLD [1,2]. DM is a complex chronic metabolic disease associated with type 1 diabetes (T1DM; juvenile-onset diabetes mellitus and insufficient insulin production) and type 2 diabetes (T2DM; insulin resistance) [3]. DM may cause damage to the blood vessels and various organs, such as kidneys and eyes [4,5]. NAFLD is a sign of obesity-related metabolic syndrome. If T2DM is associated with NAFLD, it will become very complex and make diabetes management more difficult. DM appears to promote the development of NAFLD and elevate the risk of cirrhosis and liver cancer [6]. Although antidiabetic agents have been developed to date, most drugs for the T2DM display side effects, including hyperglycemia, leukopenia, sore throat, hemolytic anemia, indigestion, ketoneuria, or lactic acidosis [7,8]. Eventually, patients are at risk of life-threatening challenges, high medical costs, or lowered quality of life [9]. Therefore, the development of safe treatment materials without side effects and drug resistance is required. T2DM causes susceptibility to NAFLD and advancement through insulin resistance and hyperglycemia [10].

Accumulating evidence from clinical trials and animal studies has revealed that T2DM and NAFLD are closely related to gut microbiota [11,12,13]. Environmental factors, such as diet, can not only directly control weight and metabolism but can also affect intestinal microbial group profiles. A high-fat diet can cause imbalance by changing the intestinal microbial group of mice. Decrease in beneficial probiotics and increase in pathogenic bacteria are associated with the occurrence of metabolic disorders, including T2DM, NAFLD, and hyperglycemia [14]. Depending on their composition, the gut microbiota exhibit both disease-promoting effects and protective properties for their host body. Gut eubiosis plays an vital role in glucose metabolism and immune regulation [15]; however, gut dysbiosis causes the formation of abnormal intestinal metabolites and also disrupts the intestinal barrier, which in turn facilitates intestinal bacteria and their harmful metabolites to enter the circulatory system of the host [16,17]. This abnormal entry may cause injury to various organs by disturbing insulin sensitivity, glucose metabolism, and immune homeostasis [18]. T2DM and NAFLD patients have shown imbalanced gut microbial community structure and diversity compared to healthy individuals [19,20,21]. Recent studies have revealed that consumption of probiotics can recover gut microbiota and mitigate hyperglycemia in animals and humans [22]. Probiotics are live microorganisms that are claimed to provide health benefits when consumed and are “Generally Recognized as Safe” (GRAS) [23]. Some lactobacilli strains inhibited α-glucosidase activity, lowered blood glucose content in mice, and increased insulin sensitivity [24]. Previous reports have revealed probiotics not only prevent the growth of intestinal pathogens but also regulate blood sugar through glucose metabolism [25]. Currently, probiotics are trending, with emphasis on their effects on the gut microbiome and host body [26].

In our previous study, we obtained probiotic candidates exhibiting antihyperglycemic effect via in-vitro inhibitory activity of α-glucosidase and α-amylase and selected a potential probiotic strain, *L. fermentum* MG4295 [27]. This study aimed to demonstrate *L. fermentum* MG4295 as a novel probiotic strain and evaluate its antihyperglycemic mechanisms in a high-fat diet and sugar-water-induced mouse model.

## 2. Materials and Methods

### 2.1. L. fermentum MG4295 Cultivation

The probiotic strain *L. fermentum* MG4295, which was isolated from human, was provided by MEDIOGEN Co., Ltd. (Jecheon, Korea). *L. fermentum* MG4295 was cultivated in de Man, Rogosa, and Sharpe broth (MRS, Difco, Detroit, MI, USA) at 37 °C for 15 h. The GenBank accession number for the 16S rRNA gene sequence of strain MG4295 is MW404503 as assessed in the National Center of Biotechnology Information (NCBI).

### 2.2. Evaluation of Food Safety of the Strain L. fermentum MG4295

To evaluate food safety of the strain *L. fermentum* MG4295, the following tests were performed.

#### 2.2.1. Survival Rate under Simulating Gastrointestinal Tract (GIT)

To simulate gastric juice, acidic pH conditions were performed by Mainville et al. methods [28]. Concisely, the harvested cells were centrifuged for 5 min (3750× *g* at 4 °C) after culture for overnight and discard supernatant. Next, pellets were washed with PBS (pH 7.0) and adjusted to 10^8^ CFU/mL. To test for resistance to pepsin and pancreatin, *L. fermentum* MG4295 was suspended in simulated gastric fluid (pH 2, 3, and 4 with 1 N HCl) and simulated intestinal fluid (pH 7 and 8 with 1 N NaOH). The cell suspensions were cultivated at 37 °C at 3–4 h. The survival rate of strain in each condition was evaluated by observing viable cells. The strain viability was decided by counting colonies on MRS agar plate using colony-forming units per ml (CFU/mL).

#### 2.2.2. Auto-Aggregation

To investigate the capability of *L. fermentum* MG4295 to adhere to intestinal cells indirectly, an auto-aggregation was conducted according to Kos et al. [29]. The cell culture of the strain was inoculated (2%, *v*/*v*) in MRS broth and cultivated at 37 °C for overnight. Then, pellets were obtained by centrifuge (4000× *g*, 4 °C, and 15 min). After resuspending *L. fermentum* MG4295 in buffered saline (pH 7.0) to a final concentration of 1.0 at OD_600_, 4-mL aliquots (0.1 mL of *L. fermentum* MG4295 resuspension with 3.9 mL PBS) were taken and vortexed for 10 s. Auto-aggregation (%) was calculated as the rate of change according to the time (5 h at one-hour intervals) compared to the initial number of viable cells.

#### 2.2.3. Antibiotic Susceptibility

Antibiotic susceptibility of *L. fermentum* MG4295 was demonstrated by the minimum inhibitory concentration (MIC) strip (Liofilchem, Via Scozia, Abruzzi TE, Italy). The antibiotic susceptibility of the strain to ampicillin, chloramphenicol, clindamycin, erythromycin, gentamicin, kanamycin, streptomycin, and tetracycline was tested according to the European Food Safety Authority (EFSA) guidelines [30]. The strain was cultivated at 37 °C for 18 h in MRS medium. Then, cell pellets were obtained by centrifuge (3750× *g*, 4 °C, 5 min) and resuspended in PBS (pH 7.4) to a McFarland standard of 0.5. The suspended strain was inoculated on BHI agar. Next, the plates with MIC test strips were incubated at 37 °C, and growth inhibition was confirmed after 48 h. The cut-off values for different antibiotics were measured by the EFSA guidelines [30].

#### 2.2.4. Hemolytic Activity

For the hemolysis test, *L. fermentum* MG4295 was plated on tryptic soy agar (Difco, Detroit, MI, USA) containing 5% sheep blood (MB cell, Seoul, Korea) and incubated for 18 h at 37 °C in a CO_2_ incubator [31]. After incubation, the plates were observed for the product of a greenish zone (α-hemolysis) and no such zone (γ-hemolysis) around the colonies.

#### 2.2.5. Enzyme Production

To evaluate enzyme activity, the *L. fermentum* MG4295 was estimated by API ZYM system with colonies as described by the manufacturer’s manual (bioMérieux, Marcy-l’Étoile, France). The enzyme production was confirmed to the strong degree of color from 0 (no activity) to 5 (≥40 nM of product released) at 10 nM intervals.

### 2.3. Sample Preparation and Animals Experimental Design

To prepare the MG4295 sample, the harvested bacterial cell pellet (obtained after centrifugation at 4000× *g*, 10 min, 4 °C) was combined with lyoprotectant cocktail and then freeze-dried [32].

Seven-week-old male C57BL/6 mice were obtained from DooYeol Biotech (Seoul, Korea). All mice were caged under climate-controlled conditions (55 ± 5% humidity, 23 ± 2 °C, and 12-h day/night cycle), fed normal diet (ND) chow (14% fat, 21% protein, and 64% carbohydrate), and supplied with water ad libitum. The animal experiments were tested by the Food and Nutrition Laboratory (FNLab; No. 20-FNL-504), and every exertion was made to minimize pain of the mice. The mice were distributed into three groups at random (*n* = 8 per group; ND, high-fat diet (HFD) and HFD plus *L. fermentum* MG4295). The HFD group was fed chow containing 60% fat and drinking water (55% fructose + 45% sucrose, 42 g/L). In the HFD plus *L. fermentum* MG4295 group, probiotic powder was prepared in sterilized PBS (pH 7.4) and orally administered (1 × 10^9^ CFU/mouse/day) for 12 weeks. At the end of the study, all animals were food deprived for 12 h and anesthetized by CO_2_ inhalation and euthanized by cardiac puncture. Liver, muscle, and adipose tissues were rapidly excised and fixed in formalin or stored at−80 °C until analysis.

### 2.4. Evaluation of Weight of Body and Tissue

Weight of body was measured each 12 weeks. Diets were fed to mice simultaneously. The liver and adipose tissues were dissected, washed with PBS, and weighed. The dissected tissues were stored in a deep-freezer until further experiments.

### 2.5. Measurement of Oral Glucose Tolerance Test (OGTT)

The animals were subjected to an OGTT during the week prior to euthanasia. Briefly, all mice were fasted for 12 h and administered glucose (2 g/kg body weight) by oral route; blood glucose levels were measured at 0 and 15, 30, 60, 90, and 120 min after administration of the glucose load. Area under the curve (AUC) of the BGL was analyzed to glucose tolerance.

### 2.6. Biochemical Analysis and Incretin Hormone Concentration

Blood was gathered in EDTA-coated tubes, and the serum was collected by centrifuging blood sample at 3000 rpm for 20 min at 4 °C. Alanine aminotransferase (ALT), aspartate aminotransferase (AST), total cholesterol (TC), and glucose levels in serum were analyzed by a biochemical analyzer (Hitachi, Tokyo, Japan). Low-density lipoprotein cholesterol (LDL) was evaluated using LDL-Cholestest Kit (SEKISUI MEDICAL Co., Ltd., Tokyo, Japan). Glucagon-like peptide-1 (GLP-1) was determined in the serum using a GLP-1 Multispecies ELISA Kit (Invitrogen, Waltham, MA, USA) and absorbance reader (800TS, BioTek, Winooski, VT, USA).

### 2.7. Histopathological Examination and Diameter of Adipocytes

The effect of *L. fermentum* MG4295 on steatosis was measured by histopathological changes in the tissues of liver and/or adipose. The tissues were fastened in 10% formalin for at least 24 h, and subsequently, 4-μm liver and adipose tissue sections were obtained and were stained with hematoxylin and eosin (H&E). Histopathological changes in the liver tissues were expressed by the summation of four grades as follows: 0 = normal, 1 = minimal, 2 = moderate, and 3 = severe.

### 2.8. RNA Extraction and Quantitative Real-Time Polymerase Chain Reaction (qRT-PCR)

Total RNA in liver or muscle tissue was extracted by NucleoZOL (MACHEREY-NAGEL, Duren, Germany), and cDNA was synthesized using first strand Reverse Transcription Premix kit (Elpis Biotech Co. Ltd., Daejeon, Korea) according to the manufacturer’s instructions. qPCR amplification was executed by CFX ConnectTM Real-Time System (Bio-Rad, Hercules, CA, USA) using iQTM SYBR^®^ Green Supermix (Bio-Rad) with cycle conditions as follows: pre-denaturation 95 °C for 3 min, denaturation 95 °C for 10 s, annealing 50–65 °C for 30 s, followed by 39 amplification cycles; melt curve was 65 to 95 °C for 0.05 s. The target genes were insulin receptor substrate-1 (*IRS-1*), protein kinase B (*AKT*), and glucose transporter-4 (*GLUT4*) for insulin signaling pathways and glucose-6-phosphatase catalytic subunit (*G6Pc*) and phosphoenolpyruvate carboxykinase (*PEPCK*) for gluconeogenesis. The primer sequences used for expression analyses of the mouse genes primers are indicated (Table S1). The target genes expression was normalized to GAPDH and calculated using the 2^−^^△△^^Ct^ method [24,33,34].

### 2.9. Statistical Analysis

All data are expressed as mean ± standard deviation (SD) of triplicate determinations. The statistical significance of differences was calculated using a one-way analysis of variance (ANOVA) using Duncan’s multiple range test, and *p* < 0.05 was regarded to be statistically significant using the SPSS (Statistical Package for the Social Sciences, ver. 21.0, Chicago, IL, USA).

## 3. Results

### 3.1. Evaluation of Food Safety of the Strain L. fermentum MG4295

#### 3.1.1. Survival of the Strain *L. fermentum* MG4295 under Conditions Simulating the Human Gastrointestinal Tract and Adhesion Ability

*L. fermentum* MG4295 was exposed to simulated gastric fluid and intestinal conditions. Simulated gastrointestinal conditions showed cell viability from 4.5 ± 0.03 to 7.6 ± 0.04 log CFU/mL (Table 1). These results indicate that *L. fermentum* MG4295 can survive in gastrointestinal conditions of the host body. Among the pH conditions, *L. fermentum* MG4295 showed a lower growth rate in the pH 2 condition than in other pH conditions. However, there is a need to improve the survivability by performing culture optimization or applying cell-coating technology. The adhesion ability of the strain MG4295 was 58.0 ± 11.4% of the aggregation ability at 5 h.

#### 3.1.2. Antibiotic Susceptibility and Hemolysis

To demonstrate food safety, *L. fermentum* MG4295 was investigated for antibiotic resistance using the MIC and hemolytic tests (Table 2). The strain was within the epidemiological cut-off values suggested by the EFSA (2018). The strain showed neither alpha nor beta hemolysis. However, it showed gamma hemolysis activity as a negative response (data not shown).

#### 3.1.3. Enzyme Production

The enzymatic activity of *L. fermentum* MG4295 was evaluated using an API ZYM system (Table 3). *L. fermentum* MG4295 did not produce alkaline phosphatase, lipase, crystine arylamidase, trypsin, α-chymotrypsin, acid phosphatase, β-glucuronidase, β-glucosidase, N-acetyl-β-glucosaminidase, α-mannosidase, valine arylamidase, naphthol-AS-B1-phosphohydrolase, α-fucosidase, or α-glucosidase. In particular, β-glucuronidase is a bacterial carcinogenic enzyme that shows negative effects on the liver [21].

### 3.2. Weight of Body and Tissues

**Figure 1:**
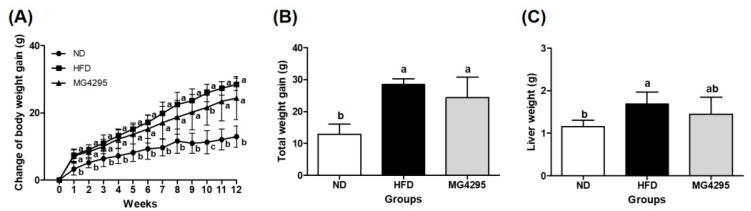
Effect of the *L. fermentum* MG4295 on (**A**) change in body weight gain, (**B**) total weight gain, and (**C**) liver weight gain in HFD-induced mice for 12 weeks. The results are presented as mean ± SD (*n* = 8). Different letters indicate a significant difference at *p* < 0.05.

To evaluate the change in weight, we measured the body weight of mice and tissue weight of the liver and adipose tissue (Figure 1A; Table S2). The HFD group exhibited significantly higher body, liver, and adipose tissue weights than the ND group (*p* < 0.05) (Figure 1A,C; Table S2). The *L. fermentum* MG4295-treated group did not show a change in the weight of adipose tissues compared to HFD group (Table S2). Although there were no significant differences, the *L. fermentum* MG4295-treated group showed reduced body weight (14.3%) and liver weight (13.9%) than HFD group (Figure 1B,C).

### 3.3. Effect of L. fermentum MG4295 on OGTT

**Figure 2:**
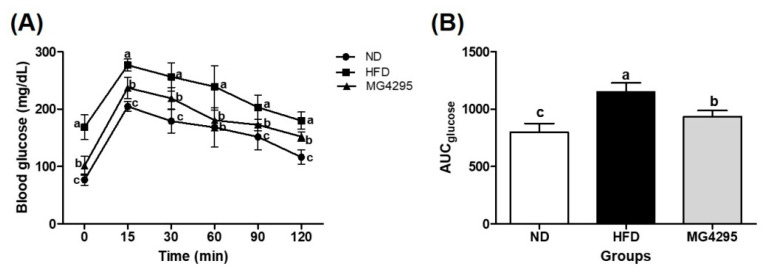
Effect of *L. fermentum* MG4295 on (**A**) OGTT and (**B**) AUC in HFD-induced mice. The results are presented as mean ± SD (*n* = 8). Different letters indicate a significant difference at *p* < 0.05.

After 12 weeks of administration, an OGTT was performed to evaluate glucose tolerance (Figure 2). The HFD-induced group showed greater glucose content represented as AUC after oral glucose loading and glucose tolerance test compared to the ND group (*p* < 0.05) (Figure 2A). Moreover, administration of *L. fermentum* MG4295 caused a significant reversal of the disrupted glucose homeostasis induced by the HFD diet (Figure 2B).

### 3.4. Effect of L. fermentum MG4295 on Serum and Incretin Hormone Concentrations

**Figure 3:**
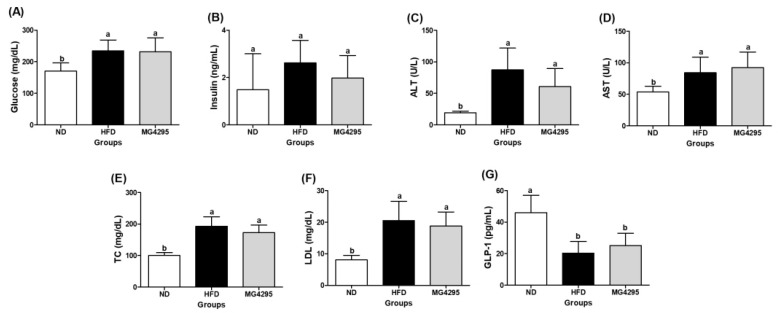
Effect of *L. fermentum* MG4295 on biochemical parameters. (**A**) Glucose, (**B**) insulin, (**C**) ALT, (**D**) AST, (**E**) TC, (**F**) LDL, and (**G**) GLP-1 in HFD-induced mice serum. The results are presented as mean ± SD (*n* = 8). Different letters indicate a significant difference at *p* < 0.05.

At the end of the experiment, the HFD-induced group exhibited increased serum levels of glucose, insulin, ALT, AST, TC, LDL, and GLP-1 (Figure 3). Although the *L. fermentum* MG4295-treated group did not display a significant difference with the HFD-induced group, administration of *L. fermentum* MG4295 led to reduced insulin, ALT, TC, and LDL levels and increased GLP-1 levels compared to HFD-induced group.

### 3.5. Histopathological Examination of The Liver and Diameters of Adipocytes in Epididymal Adipose Tissues

**Figure 4:**
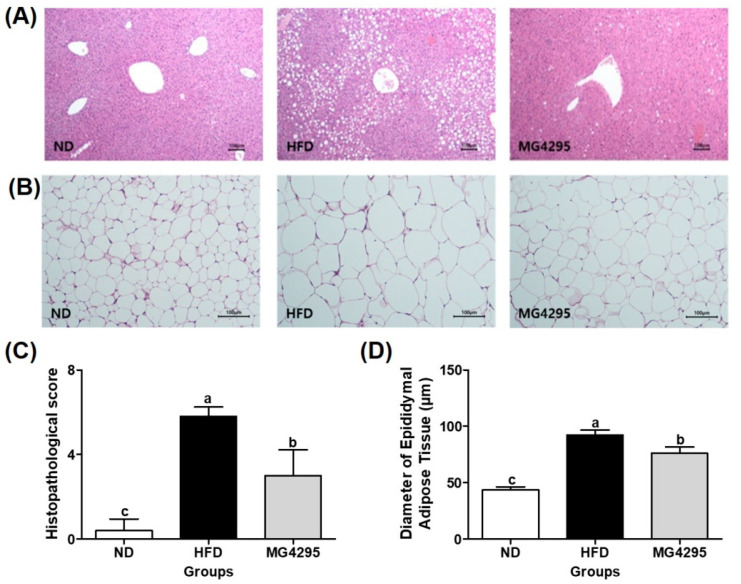
Histopathological alterations in the liver and epididymal adipose tissues. Representative photographs of H&E-stained liver tissues (**A**) and epididymal adipose tissues (**B**), histopathological scores of liver tissues (**C**), and diameters of epididymal adipose tissues (**D**) in HFD-induced mice. Scale bars = 100 μm. The results are presented as mean ± SD (*n* = 8). Different letters indicate a significant difference at *p* < 0.05.

Histological changes in liver and adipose tissues were assessed to confirm improvement due to the administration of *L. fermentum* MG4295 (Figure 4A,B). In the HFD-induced group, the histopathological total score (5.8 ± 0.5), including the level of steatosis, lobular inflammation, and hepatocellular hypertrophy, was markedly increased compared to that in the ND (0.4 ± 0.6) group. In contrast, in the *L. fermentum* MG4295-treated group, the score was significantly reduced (3.0 ± 1.2) compared to that in HFD group (Figure 4C). Regarding the diameters of adipocytes in the adipose tissue, hypertrophy (increases of mean diameters) of epididymal adipose cells was more markedly observed in HFD-induced group (92.2 ± 4.5 μm) than in ND group (43.5 ± 2.8 μm) (Figure 4D). However, it was decreased in the *L. fermentum* MG4295 group (76.1 ± 5.6 μm) compared to the HFD-induced group. In this study, *L. fermentum* MG4295 showed an improvement in the histopathological score in liver tissues and lipid accumulation in adipocytes.

### 3.6. Effect of L. fermentum MG4295 on Regulation of Insulin and Gluconeogenesis Signaling Pathway

**Figure 5:**
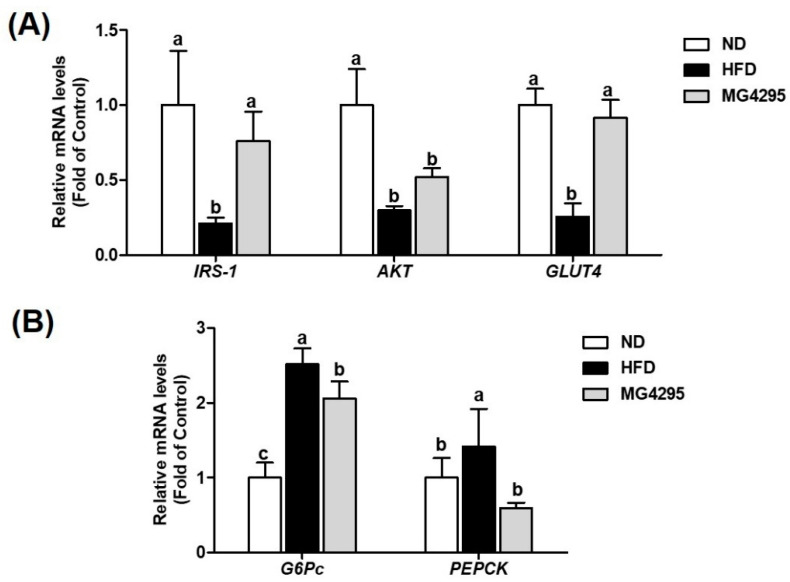
Effect of *L. fermentum* MG4295 on (**A**) insulin signaling (*IRS-1* and *AKT* in liver tissues and *GLUT*4 in muscle tissues) and (**B**) gluconeogenesis (*G6Pc* and *PEPCK*) in liver-related mRNA expressions in HFD-induced mice. The results are presented as mean ± SD (*n* = 8). Different letters indicate a significant difference at *p* < 0.05.

The effect of *L. fermentum* MG4295 on insulin and gluconeogenesis signaling pathway-related gene expression in the liver and muscle via qPCR was assessed (Figure 5). Compared to the ND group, the HFD-induced group exhibited significantly downregulated the mRNA expression of *IRS-1*, *AKT*, and *GLUT4* (0.21-, 0.30-, and 0.26-fold of control, respectively, *p* < 0.05) and upregulated the mRNA expression of *G6Pc* and *PEPCK* (2.52- and 1.42-fold of control, respectively). Conversely, administration of *L. fermentum* MG4295 significantly upregulated *IRS-1* and *GLUT4* mRNA expression (0.76- and 0.91-fold of control, respectively), and *G6Pc* and *PEPCK* mRNA expression was significantly decreased (2.06- and 0.59-fold of control, respectively). For HFD-induced group and *L. fermentum* MG4295-treated group in *AKT* (0.52-fold of control), mRNA expression showed no significant difference. In *AKT* mRNA expression, the *L. fermentum* MG4295 group increased that of levels.

## 4. Discussion

In recent years, probiotics have attracted the attention of scientists as functional foods to be used as dietary supplements and for preclinical and/or clinical trials [35]. Although probiotics are defined as GRAS or belong to species qualified presumption of safety status, novel candidates have to establish this qualification to be used as probiotics. In this regard, prerequisites, such as stability, safety, and health benefits, have been investigated. In stability study, *L. fermentum* MG4295 survived in simulated gastrointestinal conditions; the percentage viability each gastric and intestinal samples ranged from 58.9 to 98.9% and 97.7 to 99.4%, respectively. A low pH of gastric juice is one of the important health benefits to inhibit the overgrowth of pathogens that induce gut dysbiosis [36]. However, as an acidic pH destroys many microorganisms that are ingested, resistance to human gastric transit is a major selection criterion for microorganisms present in probiotics [37]. Auto-aggregation is one of the important qualities of probiotics for colonizing epithelial cells [38,39]. Our strain showed relatively high auto-aggregation values (58%) compared to the strains used in other studies (<30%) [40,41]. In this respect, *L. fermentum* MG4295 is likely to survive in the gastrointestinal condition, as it showed survivability and adhesive ability in the stomach and intestinal conditions. The safety of probiotics is important for the choice as probiotic strains and consumer demand [42,43]. Thus, the safety and functionality of probiotics should be evaluated using global standards. In our study, *L. fermentum* MG4295 showed no hemolysis or β-glucuronidase activity. Additionally, if probiotics have antibiotic resistance, those probiotics could transfer antibiotic resistance to pathogenic bacteria that can cause serious problems [43,44]. Lower cut-off MIC values for the antibiotics test of *L. fermentum* MG4295 fulfilled global standards. In conclusion, these results demonstrate that *L. fermentum* MG4295 could be used as a probiotics.

Supplementation with *L. fermentum* MG4295 showed a tendency to cause loss of body and liver weight in the HFD group, suggesting that *L. fermentum* MG4295 could prevent obesity and ameliorate hyperglycemia. Previous studies have reported that probiotic dairy products containing *L. acidophilus* and *L. casei* could defer the beginning of glucose intolerance and hyperglycemia in a high fructose-induced rat model [45]. Moreover, the consumption of probiotic product, including *L. acidophilus* La5 and *Bi. lactis* Bb12, restored the fasting blood glucose in T2DM patients [46]. Furthermore, treatment with *L. plantarum* NA136 significantly reduced weight gain and adipose tissue, lipids, AST, and ALT levels in HFD/F-treated mice [47]. In our study, administration of *L. fermentum* MG4295 for 12 weeks improved diabetic, NAFLD parameters, and oral glucose tolerance in HFD-induced groups.

GLP-1 is a kind of incretin secreted by intestinal enteroendocrine cells [48]. GLP-1 has many physiological effects, such as stimulation of glucose-dependent insulin secretion and increment of B-cell numbers [49,50]. Many studies have described that the probiotics elevate the release of GLP-1 and decrease inflammation and IR caused by HFD [33,51,52]. This hormone affects the GIT by inhibiting the secretion of gastric juice and gastrointestinal peristalsis, delaying the emptying of gastric contents, and stimulating the hypothalamus in the central nervous system to increase the sense of satiety and appetite [53,54]. Thus, weight, fasting blood glucose level, and other indicators related to T2DM can be decreased [52]. Our results are consistent with previous studies that revealed a hyperglycemic effect in HFD-induced groups.

In T2DM, insulin resistance can be defined as a metabolic state with a relatively low insulin action (insulin sensitivity) compared to the normal state of physiological insulin concentrations [4,8]. IR appear in target organs as well as liver, muscles, and adipose fat tissues [7]. IR causes compensatory hyperinsulinemia along with metabolic abnormalities, such as glucose hypersensitivity, hyperlipidemia, hepatic steatosis, and NAFLD [55]. The insulin signaling pathway plays a key role in controlling IR and glucose metabolism [56]. To elucidate the putative mechanisms of *L. fermentum* MG4295 on insulin signaling pathways and gluconeogenesis in HFD-induced groups, target genes were investigated. The PI3K was initiated by tyrosine phosphorylation of the IRS-1 in reaction to insulin stimulation [57]. Activation of PI3K catalyzes the phosphorylation of Akt that activate downstream proteins containing glycogen synthesis [9]. Eventually, these stages are activated, and GLUT4 from the plasma membrane of myocytes is translocated to enhance glucose transport [34]. In our study, factors related to insulin signaling pathway, including *IRS-1*, *AKT*, and *GLUT4*, were elevated by the administration of *L. fermentum* MG4295 to HFD-induced mice. Additionally, *G6Pc* and *PEPCK* play major roles in gluconeogenesis stimulated by glucagon [58]. In a previous study, *L. paracasei* CCFM0416, *L. fermentum* MTCC 5689, and *L. plantarum* MTCC 5690 suppressed the expression of *G6Pc* and *PEPCK* in liver tissues [24,33]. Similarly, our results showed a more significantly reduced expression of *G6Pc* and *PEPCK* involved in gluconeogenesis in the MG4295 group than in HFD group. Taken together, this study suggests that *Limosilactobacillus fermentum* MG4295 could be usable as a functional food and nutraceutical to prevent diabetes by modulating the insulin and gluconeogenesis signaling pathways.

## 5. Conclusions

In this study, *L. fermentum* MG4295 was verified to be a probiotic by investigating its properties and safety. *L. fermentum* MG4295 improved serum profiles, including glucose, insulin, ALT, TC, LDL, HDL, and GLP-1 levels of HFD-induced mice. It also significantly reduced weight gain, hepatic histopathological score, and diameters of adipocytes of epididymal adipose tissues. Furthermore, *L. fermentum* MG4295 effectively ameliorated IRS-1, AKT, GLUT4, G6Pc, and PEPCK expressions. This result means that *L. fermentum* MG4295 could be regulate insulin and gluconeogenesis pathways. Consequently, *L. fermentum* MG4295 can be used as a probiotic strain to improve hyperglycemia and NAFLD.

## Figures and Tables

**Table 1:** Survivability of the probiotic candidate *L. fermentum* MG4295 under simulated GIT.

Strain	Initial	Simulated Gastric Fluid ^a^			Simulated Intestinal Fluid ^b^	
		PH 2	PH 3	PH 4	PH 7	PH 8
*L. fermentum* MG4295	7.6 ± 0.04	4.5 ± 0.03	7.1 ± 0.05	7.5 ± 0.07	7.5 ± 0.02	7.6 ± 0.04
		(58.9%)	(93.3%)	(98.9%)	(97.7%)	(99.4%)

The results are expressed as the mean ± SD (*n* = 3). All values are shown as viable counts (log CFU/mL). ^a^ Simulated gastric tolerance results are pH 2, pH 3, and pH 4 at 37 °C after 3 h. ^b^ Simulated intestinal tolerance results pH 7 and pH 8 at 37 °C after 4 h.

**Table 2:** Results of minimum inhibitory concentration test for the probiotic candidate *L. fermentum* MG4295.

Antibiotics (μg/mL)	*L. fermentum* MG4295	
	MIC	EFSA
Ampicillin	0.125	2
Chloramphenicol	3	4
Clindamycin	0.016	4
Erythromycin	0.064	1
Gentamicin	0.125	16
Kanamycin	3	64
Streptomycin	1.5	64
Tetracycline	4	8

MIC, minimum inhibitory concentration; EFSA, EFSA cut-off value [26].

**Table 3:** Enzymatic activity profiles of the probiotic candidate *L. fermentum* MG4295.

Enzyme	Activity
Esterase	3
Esterase Lipase	4
Leucine arylamidase	4
α-Galactosidase	5
β-Galactosidase	5

## Data Availability

Data are available in a publicly accessible repository/data are contained within the article or Supplementary Materials.

## Supplementary Materials

The following are available online at https://www.mdpi.com/article/10.3390/foods11020231/s1, Table S1: Primers sequence used for qRT-PCR; Table S2: Liver and adipose tissues weight.
