# Supplementary material for: Limosilactobacillus fermentum MG4295 Improves Hyperglycemia in High-Fat Diet-Induced Mice
Source: Foods. 2022 Jan 15;11(2):231. doi: 10.3390/foods11020231 (PMC8774940; doi:10.3390/foods11020231)
Supplement: Supplementary file 1 [file foods-11-00231-s001.zip › foods-1542012-supplementary.pdf]

Table S1. Primers sequence used for qRT-PCR.

Gene name	Forward 5´to 3´	Reverse 5´to 3´
IRS-1	AAGGAGGTCTGGCAGGTTATC	ATGGTCTTGCTGGTCAGGC
AKT	ACTCATTCCAGACCCACGAC	CCGGTACACCACGTTCTTCT
GLUT4	CATTCCCTGGTTCATTGTGG	GAAGACGTAAGGACCCATAGC
G6Pc	TCTTGTGGTTGGGATTCTGG	CGGATGTGGCTGAAAGTTTC
PEPCK	CCATCCCAACTCGAGATTCTG	CTGAGGGCTTCATAGACAAGG
GAPDH	CACTCACGGCAAATTCAACGGCA	GACTCCACGACATACTCAGCAC

IRS-1, insulin receptor substrate-1; *AKT*, protein kinase B; *GLUT4*, glucose transporter-4; *G6Pc*, glucose-6-phosphatase catalytic subunit; *PEPCK*, phosphoenolpyruvate carboxykinase; *GAPDH*, glyceraldehyde 3-phosphate dehydrogenase.

Table S2. Liver and adipose tissues weight.

Groups	Liver	Epididymal	Mesenteric	Perirenal	Retroperitoneal	Subcutaneous
ND	1.2 ± 0.2	0.4 ± 0.05	0.4 ± 0.1	0.03 ± 0.02	0.2 ± 0.1	0.3 ± 0.1
HFD	1.7 ± 0.3	1.9 ± 0.4	1.4 ± 0.4	0.3 ± 0.1	0.7 ± 0.1	2.0 ± 0.6
MG4295	1.5 ± 0.4	2.1 ± 0.6	1.3 ± 0.6	0.3 ± 0.1	0.8 ± 0.2	1.8 ± 0.6

Data are presented as mean ± SD (n = 8).
